# Associations of common variants at *ALDH2* gene and the risk of stroke in patients with coronary artery diseases undergoing percutaneous coronary intervention

**DOI:** 10.1097/MD.0000000000010711

**Published:** 2018-05-11

**Authors:** Ling You, Chenze Li, Jinzhao Zhao, Dao Wen Wang, Wei Cui

**Affiliations:** a Division of Cardiology, The Second Hospital of Hebei Medical University, Shijiazhuang, Hebei Province; b Departments of Internal Medicine and Genetic diagnosis Center, Tongji Hospital, Tongji Medical College, Huazhong University of Science and Technology, China.

**Keywords:** clinical outcome, coronary heart disease, genetic

## Abstract

Limited data are available about the role of common variants at the *aldehyde dehydrogenase 2* gene (*ALDH2*) on the clinical outcome in Chinese patients with coronary heart disease (CHD) undergoing percutaneous coronary intervention (PCI). In the present study, a total of 1089 patients were consecutively enrolled from January 2012 and July 2013. Six common variants at *ALDH2* gene, including rs2339840, rs4648328, rs4767939, rs11066028, rs16941669, and rs671, were selected to test the associations of those polymorphisms with the cardiovascular outcome in patients with CHD after PCI. The clinical endpoints included cardiovascular death, nonfatal myocardial infarction, and nonfatal stroke. The composite of clinical endpoints was defined as the primary endpoint, and every endpoint alone was considered as the secondary endpoints. The median follow-up time was 38.27 months. Our results showed that the common variant rs2339840 was independently associated with a lower risk of stroke in patients with CHD after PCI (codominant model, HR = 0.32, 95% CI, 0.11–0.91, *P* = .074 for heterozygotes; HR = 0.25, 95% CI, 0.06–1.14, *P = *.033 for homozygotes; dominant model, HR = 0.32, 95% CI, 0.14–0.74, *P = *.007). However, no significant associations were found between other 5 single nucleotide polymorphisms (SNPs) and the clinical endpoints. For the first time, the common variant rs2339840 was reported to be a protective factor against stroke in CHD patients with PCI.

## 1 Introduction

Coronary heart disease (CHD) is the leading cause of death in the worldwide.^[1]^ For now, CHD is recognized as a complex disease and characterized by an interplay between genetic and environment.^[2]^ In the past decades, with the increasing advancement in the field of genome technology, numerous genetic variants have been identified to be related with the morbidity and mortality of CHD by genome wide association study (GWAS) and the next generation sequencing (NGS).^[3]^ Such as, *aldehyde dehydrogenase 2* (*ALDH2*) gene was identified as one of the candidate gene that associated with an increased risk of CHD.^[4,5]^ We previously focused on the association of common variants at *ALDH2* gene with the occurrence of CHD. The findings showed that the common variants rs671 at *ALDH2* gene were associated with an increased risk of CAD in South Chinese, while not in North Chinese.^[6]^ In the present study, our primary objective was to explore whether the single nucleotide polymorphisms (SNPs) at *ALDH2* gene were associated with the cardiovascular outcome and its individual endpoints, and discussed its possible mechanism.

## 2 Methods

### 2.1 Study subjects

In the present study, we consecutively recruited patients from the Tongji Hospital in Wuhan (Hubei, People's Republic of China). The inclusion criteria included: Patients were diagnosed as CHD by professional cardiologists, and confirmed by coronary angiography that >50% diameter stenosis in at least 1 coronary artery during coronary angiogram; undergoing successful PCI. Additionally, following patients were excluded from our study: Patients were younger than 18 years old or older than 80 years old; in-hospital death; refusal to participate in the study; blood samples could not be available. Finally, from January 2012 and July 2013, a total of 1089 patients with coronary heart disease undergoing PCI were enrolled. The study protocol was approved by local ethics committee and conducted according to the Declaration of Helsinki Guidelines for Good Clinical Practice. Written informed consent was obtained from each participant.

### 2.2 Follow-up and clinical outcome

The baseline characteristics, including demographics, physical examination, diseases history, treatment during in-hospital, and laboratory test, were recorded by 2 independent investigators. Once disagreement occurred, the primary investigator adjudicated the information. After patients were discharged, they were followed-up at regular time by cardiological nurses and their clinical outcomes, such as death, myocardial infarction, stroke, and any other rehospitalization were all recorded. In this study, cardiovascular death, myocardial infarction, and stroke were considered as the primary endpoint. Of these, cardiovascular death was defined as cardiogenic death and other unknown death, including sudden death. Myocardial infarction was identified as chest pain with obvious electrocardiographic changes as well as the elevated cardiac injury biomarkers. In addition, stroke was referred to as an acute neurological deficit lasting more than 24 hours. All of the definitions were consistent with the guideline of American College of Cardiology/American Heart Association (ACC/AHA).^[7]^

### 2.3 SNP selection and genotyping

Firstly, we searched the position of *ALDH2* gene in the University of California Santa Cruz (UCSC). To ensure the range incorporating the 5′UTR and 3′UTR, we added 2 kb nucleic acid in the right and left genomic deoxyribonucleic acid (DNA), and used the Hapmap project as the reference panel, as previously described. At last, 6 tag SNPs, including rs2339840, rs4648328, rs4767939, rs11066028, rs16941669, and rs671, were selected to represent all 16 common SNPs in this region.

Thereafter, the genomic DNA was extracted from the peripheral blood sample using a commercially DNA extraction kit, and we performed the extraction procedure according to the manufacturer's instructions. The 6 common variants were genotyped by Taqman fluorescent allelic discrimination on 7900 HT fast Real-Time PCR System (Applied Biosystems, Foster City, CA). The Taqman probes and primers were designed by ABI Primer Expression 3.0 software and synthesized by Shanghai GeneCore BioTechnologies. Co. Ltd., China. The Sequence Detection Systems 2.4 software (Applied Biosystems) was used to detect the allelic discrimination. Approximately 10% samples were randomly selected to directly sequencing by a 3130 genetic analyzer (Applied Biosystems, Foster City, CA) to confirm the quality of TaqMan SNP allelic discrimination.

### 2.4 Statistical analysis

Continuous data with the normal distribution were summarized as means ± standard difference (SD), otherwise median (interquartile range, IQR). For categorical variables, data were presented as count (percentage). Independent sample *t* test was used to compare the means ± SD between 2 groups, but Mann–Whitney test was applied to examine the difference for data with the non-normal distribution. Comparisons between categorical variables were performed by chi-square test or Fisher exact test according to their expected number. Additionally, chi-square test was also used to test whether conform to Hardy Weinberg equilibrium for genotypic distribution. To test the association of the genotype of with the clinical outcome, Kaplan–Meier method was used to describe the survival curve, and the difference between the curves was compared by the log-rank test. Furthermore, the hazard ratio (HR) was estimated by univariable Cox regression model. However, the associations might be influenced by the confounding factors. The multivariable Cox regression model was further applied to compute the adjusted HR. The variables associated with the endpoint (*P < *.05) were selected into the multivariable Cox regression model.

All statistical analyses were performed by SPSS software (version 20.0). The tests were all 2 side, and *P* value < 0.05 was considered as statistically significant.

## 3 Results

### 3.1 Population characteristics

From January 2012 and July 2013, a total of 1089 patients were enrolled into the study. In our cohort, the median age was 60 years (52–68), and the proportion of female patients was 22%. About 50% patients have a normal BMI and WHR. Besides, the level of systolic blood pressure (SBP) and diastolic blood pressure (DBP) was 133 mm Hg (interquartile range, IQR, 120–147 mm Hg), 80 mm Hg (70–89 mm Hg), respectively. Most of patients also had a normal heart rate at baseline. 473 patients (43.6%) had a history of hypertension and 894 (82.5%) had a diabetes mellitus. 490 patients (45.5) were identified as smokers, and 724 (67.5) were drinkers. Except for demographics and disease history of patients, related laboratory test, including glucose (Glu), triglyceride (TG), total cholesterol (TC), high-density lipoprotein (HDL), low-density lipoprotein (LDL), apolipoprotein A1 (ApoA1), and apolipoprotein B (ApoB) were also recorded in detail. The data were summarized in Table 1. We then divided patients into 2 groups according to the occurrence of the primary endpoint. The results showed that the patients in the event group were older than those in the nonevent group. The level of glucose was also higher in the event group. However, the level of ApoA1 was observed lower in the event group. All other characteristics were comparable between groups. Additionally, patients were also compared according to other endpoints alone, and Table 2 was used to describe the characteristics of patients.

**Table 1:**
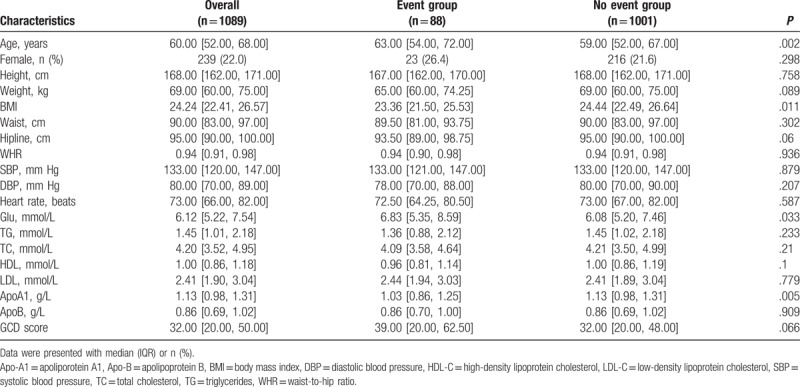
Baseline characteristics of the total population.

**Table 2:**
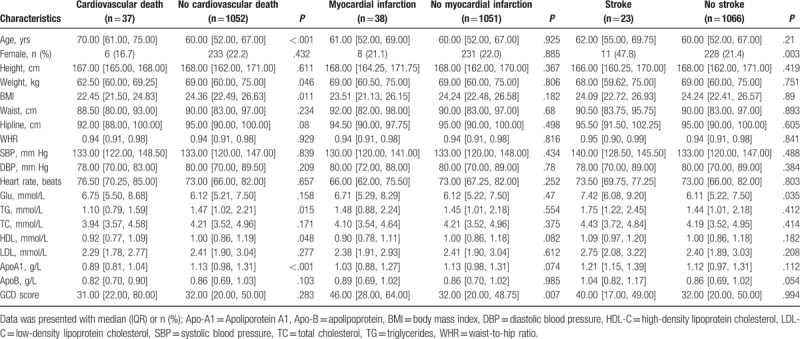
The relationship of baseline characteristics with individual endpoints.

### 3.2 Clinical outcomes

**Figure 1:**
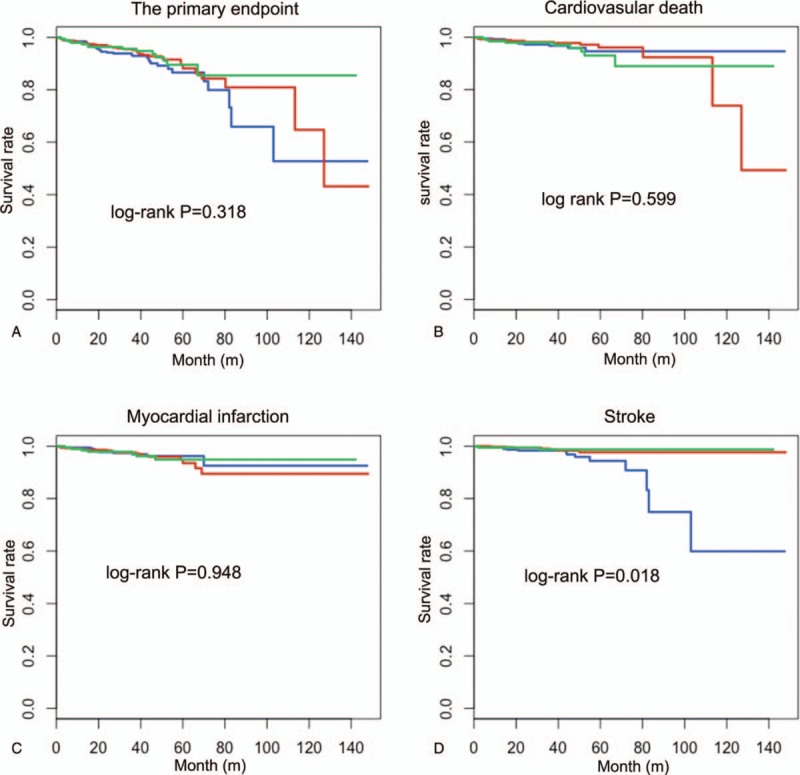
The survival curves described according to the rs2339840 genotype for the primary endpoint and the secondary endpoints. The green line represented rs2339840 homozygotes, the red line represented rs2339840 heterozygotes, and the blue line represented the wild type.

**Figure 2:**
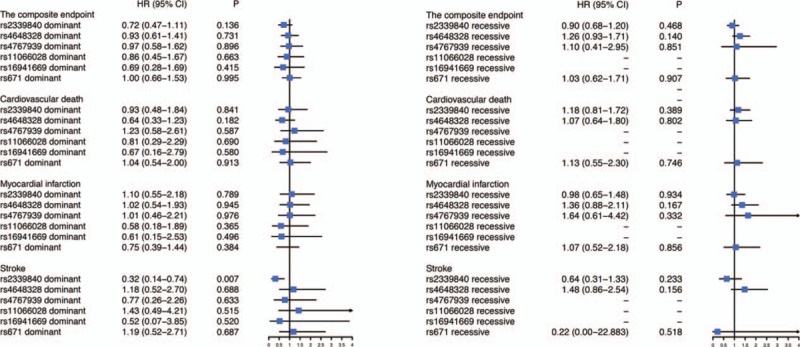
The associations of the variants with the primary endpoint and the secondary endpoints under the dominant model and the recessive model. – indicates the value could not be computed.

During the follow-up, 37 patients (3.4%) occurred cardiovascular death, 38 patients (3.5%) had a myocardial infarction, and 23 patients (2.1%) suffered from stroke. Totally, there were 88 events (8.1%) in our cohort at a median follow-up time with 38.27 months. We tested the relationship of the common variants with the primary endpoints and the secondary endpoints. The results indicated that the rs2339840 heterozygote showed a lower event rate of stroke compared with the wild type (HR = 0.32, 95% CI, 0.11–0.91, *P = *.033). Although the statistical difference was not significant between the rs2339840 homozygote and the risk of stroke because the sample size was not enough, the trend was obvious (HR = 0.25, 95% CI, 0.06–1.14, *P = *.074). The survival curves also showed the rs2339840 variant was a protective factor from the occurrence of stroke (log-rank *P = *.018) (Fig. 1). However, the associations were not significant for the primary endpoint and other individual endpoints (Table 3). Besides, the relationships between other five SNPs and the clinical outcomes were listed in Table 3. For further analysis of the effect of the variants on the cardiovascular outcome, the dominant model and the recessive model were evaluated. Similarly, the rs2339840 mutant carriers decreased the incidence of stroke in patients with CHD after PCI in the dominant model (HR = 0.32, 95% CI, 0.14–0.74, *P = *.007). The results were all summarized in Figure 2.

**Table 3:**
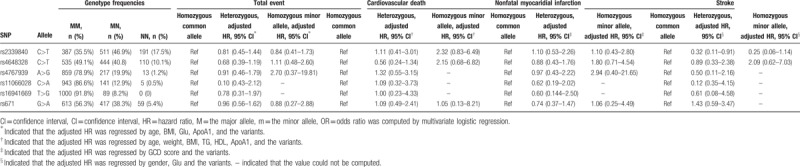
The relationships of SNPs with the clinical outcomes.

## 4 Discussion

In the present study, we investigate the relationships of the tag SNPs at the *ALDH2* gene with the clinical outcomes in patients with CHD undergoing PCI. Our results showed that the variant rs2339840 was significantly associated with a decreased risk of stroke (codominant model, HR = 0.32, 95% CI, 0.11–0.91, *P = *.074 for homozygotes; HR = 0.25, 95% CI, 0.06–1.14, *P = *.033 for heterozygotes; dominant model, HR = 0.32, 95% CI,0.14–0.74, *P = *.007). For the first time, we identified a new SNP related to the prognosis of patients with CHD after PCI.

With the advancement of technology, the sequencing of human genome becomes easier for physicians. Although GWAS and the NGS have found some SNPs or genes associated with the morbidity of coronary artery diseases,^[8–12]^ the relationships of SNPs and genes are scarcely assessed with the clinical outcome of patients with CHD, especially for the high-risk patients. Up to now, several studies have focused on the evaluation of prognosis base on baseline characteristics or hemodynamics or image of stenotic coronary arteries.^[13–17]^ However, genetic factors may also be involved in the progression of diseases.^[18]^ Therefore, genetic background should be considered as an element that included into the assessment of patients’ long-term clinical outcome.

Mitochondrial aldehyde dehydrogenase 2 (*ALDH2*) is one of the key enzymes that are essential in the metabolism of acetaldehyde and detoxification of ROS-generated aldehyde adducts.^[19]^*ALDH2* is widely expressed in the organs that require high oxygen, such as heart and brain, but also exist in the liver and lung.^[20]^ The level of ALDH in organs has a wide implication on the status of diseases.^[21]^ Previous studies have reported that the deficiency of *ALDH2* was associated with an increased risk of coronary heart disease.^[22]^ Besides, the expression of *ALDH2* also has an effect on the occurrence of cancer.^[23]^ A recent study has showed that *ALDH2* is a protective factor for ischemic stroke in Han Chinese.^[24]^ Furthermore, Guo et al^[25]^ have demonstrated that the activation of *ALDH2* pathway conferred neuroprotection by clearing 4-hydroxy-2-nonenal (4-HNE), and the *ALDH2* pathway may be a potential target of therapeutic intervention in stroke.

In this study, the common rs671 was evaluated the association with the clinical outcome, but so as other tag SNPs at *ALDH2* gene. Part of the findings is similar to our previous researches that no significant association was found between the rs671 and the main outcomes, so as in the patients after PCI. However, rs2339840 was associated with the incidence of stroke in patients undergoing PCI. rs2339840 is a downstream gene variant, and bioinformatics predicted that it may regulate the level of gene by the formation of lincRNA (http://grch37.ensembl.org/). However, the specific mechanism is still unknown now, and warrants further exploration.

Several limitations must be acknowledged upon evaluating the results of this research. Firstly, multiple comparisons may exist in our study. However, the sample size was limited so that the *P* value of .05 was considered as significant, and larger cohort is needed to replicate our findings. Second, this observational study as a secondary analysis of our previous cohort, only six SNPs were investigated at the *ALDH2* gene. Afterward, more representative SNPs would be assessed the associations with the clinical outcome, and a comprehensive genetic risk score would be used to predict the prognosis of patients undergoing PCI. Lastly, the mechanism of the association of rs2339840 with stroke was not studied by vivo and vitro experiments. This SNP was first reported to be related the progression of diseases, so further elucidation is required to explain its pathway.

## 5 Conclusions

For the first time, the present study has suggested that the variant rs2339840 was a protective factor against the occurrence of stroke for patients with CHD undergoing PCI.

## Acknowledgments

The authors thank all the study researchers.

## Author contributions

**Conceptualization:** Wei Cui, Ling You.

**Formal analysis:** Ling You, Chenze Li, Jinzhao Zhao.

**Funding acquisition:** Wei Cui.

**Investigation:** Wei Cui, Ling You, Chenze Li, Jinzhao Zhao.

**Methodology:** Wei Cui, Jinzhao Zhao, Dao Wen Wang.

**Supervision:** Dao Wen Wang.
